# Prevention of an additional surgery for regional lymphadenectomy in melanoma: rapid intraoperative immunostaining of sentinel lymph node imprint smears

**DOI:** 10.1186/1746-1596-1-32

**Published:** 2006-09-25

**Authors:** Vinod B Shidham, Richard Komorowski, Marcelle Neuberg, Alonzo Walker, Bruce H Campbell, Chung-Che Chang, William W Dzwierzynski

**Affiliations:** 1 Department of Pathology, Medical College of Wisconsin, Milwaukee, WI, USA; 2 Department of Dermatology, Medical College of Wisconsin, Milwaukee, WI, USA; 3 Department of Otolaryngology, Medical College of Wisconsin, Milwaukee, WI, USA; 4 Department of Surgery, Medical College of Wisconsin, Milwaukee, WI, USA; 5 Department of Pathology, The Methodist Hospital, Houston, TX, USA; 6 Department of Plastic Surgery, Medical College of Wisconsin, Milwaukee, WI, USA

## Abstract

**Background:**

Sentinel lymph node (SLN) biopsy is performed at many institutions and is considered a standard of care in the management of cutaneous melanoma. The discriminatory immunostaining pattern with the 'MCW Melanoma Cocktail' (a mixture of MART-1 {1:500}, Melan- A {1:100}, and Tyrosinase {1:50} monoclonal antibodies) allows intraoperative immunocytochemical evaluation of imprint smears of SLNs for melanoma metastases. Cohesive cells of benign capsular melanocytic nevi that were also immunoreactive with the cocktail do not exfoliate easily for imprint smear detection.

**Methods:**

We prospectively evaluated 73 lymph nodes (70 SLN & 3 non-SLN) from 41 cases (mean 1.8, 1 to 4 SLNs/case) of cutaneous melanoma using a rapid 17-minute immunostaining previously published protocol. The results were compared with permanent sections also immunostained with 'the cocktail'.

**Results:**

19.5%, 8/41 cases (12%, 9/73 lymph nodes) were positive for melanoma metastases on permanent sections immunostained with the 'MCW melanoma cocktail'. Melanoma metastases in 87.5% (7/8) of these cases were also detected in rapidly immunostained imprint smears, with 100% specificity and 90% sensitivity. None of the 7 SLNs from 7 cases with capsular nevi showed false positive results.

**Conclusion:**

Melanoma metastases could be detected in imprint smears immunostained with 'MCW Melanoma Cocktail' utilizing a rapid intraoperative protocol. The cohesive cells of the capsular nevi do not readily exfoliate and do not lead to false positive interpretation. In a majority of positive cases, a regional lymphadenectomy could have been completed during the same surgery for SLN biopsy and wide excision of primary melanoma site, potentially eliminating the need for an additional surgery.

## Background

Skin melanoma is the sixth most common cancer in US with an increasing rate in men [1]. Due to rapidly progressive nature of the disease, aggressive definitive therapy at earliest stage is the best option. Because of associated high morbidity and the scarcity of follow up studies evaluating its survival benefits, the role of routine performance of regional lymphadenectomy in all cases irrespective of their status is controversial [2,3]. An international multi-centre randomized prospective trial by the Intergroup Melanoma Surgical Program, reported that regional node dissection offers increased survival in subset of patients with nodal metastases (60 years of age or younger with intermediate-thickness nonulcerative melanomas) [4]. Sentinel lymph node (SLN) biopsy is recommended as a tool to identify this subset of patients, who could then undergo elective regional lymph node dissection [5]. The negative status of SLN for melanoma metastases correlates closely with the negative status of regional lymph nodes [6].

Thus, prevailing evidence supports the evaluation of SLN for melanoma metastases as the standard of care to identify subset of patients with metastases in cutaneous melanoma. It is practiced throughout the academic centers as well as regional and community hospitals with increasing frequency [2-22].

Although, it is beneficial to complete a regional lymphadenectomy during the same initial surgical procedure for 'SLN biopsy with wide excision of primary melanoma', currently the patient has to undergo regional lymphadenectomy at later date under separate anesthesia, if SLNs are positive for melanoma metastases. In the current situation the results can not be obtained intraoperatively. Preoperative or intraoperative evaluation technique would result in a cost saving with numerous other benefits [23-31].

Frozen-section examination (with or without immunohistochemical evaluation) and the morphological evaluation of imprint cytology smears are some of the methods evaluated for the intraoperative examination of SLN in cutaneous melanoma. However, the studies evaluating these methods did not demonstrate encouraging results [25-31], primarily due to lack of adequate sensitivity.

The 'MCW Melanoma Cocktail' (a mixture of MART-1 {1:500}, Melan- A {1:100}, and Tyrosinase {1:50} monoclonal antibodies) demonstrated an excellent discriminatory immunostaining pattern [32,33]. This has facilitated rapid intraoperative evaluation of SLNs for melanoma micrometastases by examining imprint smears of SLNs immunostained with the cocktail [34,35]. This was not previously possible with conventional immunomarkers such as the S-100 protein and HMB45 due to significant interference caused by non-melanoma cells such as dendritic cells and mast cells resulting in a high noise to signal ratio [33,36].

In this article we report the results on a series of cutaneous melanoma cases studied with rapid intraoperative evaluation of SLN for melanoma metastases employing imprint smears immunostained with 'MCW melanoma cocktail'.

## Materials and methods

### Patients

73 lymph nodes (70 SLNs & 3 non-SLNs) from 41 cases with clinically localized cutaneous melanoma were studied prospectively under the IRB approved protocol. The demographics are shown in table 1. As this was as a research study, intraoperative decisions for the continuation of a regional lymphadenectomy were not executed regardless of positive results with immunostained imprint smear(s) of SLNs. This was explained to the patients participating in the study as a component of the informed consent.

**Table 1:** Demographics of all the cases.

	Feature	Details
**1**	Number of case studied	Total- 41 case
		Males- 21, Females- 20
		Mean age- 56 years (14 – 48 years)

**2**	Total lymph nodes studied	73 lymph nodes (SLN- 70, non-SLN- 3*) Mean- 1.7 SLN per case (1–4 SLN per case)
		1 SLN- 16 cases
		2 SLN- 17 cases
		3 SLN- 4 cases
		4 SLN- 4 cases

**3**	Pigmented lesions as indication for SLN biopsy	Melanomas- 39
		Atypical Spitzoid nevus- 1
		Dysplastic nevus- 1

**4**	Location of primary pigmented lesion	Head and neck (15), the trunk (11), the upper extremity (6), and the lower extremity (9).

**5**	Histological type of melanomas (known in 17 cases)^¶^	Superficial spreading- 11
		Nodular- 5
		Superficial spreading with nodular- 1

**6**	Breslow thickness of primary melanoma (known in 38 patients)	Mean- 2.1 mm (range 0.7 to 8.9 mm)

*Case no. 23 (Table 2) had 5 lymph nodes (2 SLN and 3 non-SLN).

^¶ ^In most cases, the biopsy of primary lesion was performed and interpreted at outside institutions.

All patients underwent mappings and biopsies of SLNs with a wide excision of the primary site at Froedert Memorial Lutheran Hospital/Medical College of Wisconsin, Milwaukee, WI. A standard surgical protocol was used to identify the SLN [37]. The tumor bed was injected preoperatively with technetium sulfur colloid. Additionally, dermis around the lesion was injected intraoperatively with isosulfan blue dye to facilitate the visual identification of the SLN [37]. The SLNs were harvested and submitted fresh without any fixative to pathology for intraoperative and permanent section evaluation.

### Pathologic examination (Figure 1)

**Figure 1:**
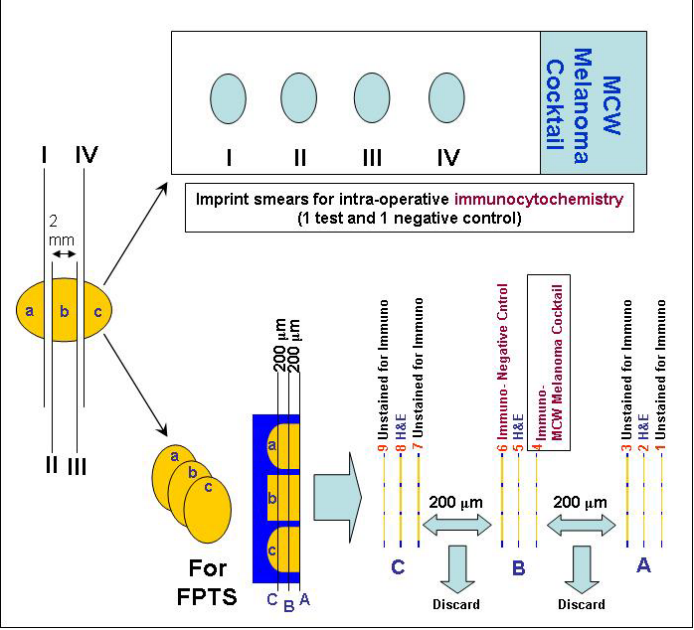
Pathological evaluation of sentinel lymph nodes for melanoma metastases. Section number 2, 5, & 8- stained with HE; 4- immunostained with 'MCW melanoma cocktail'; 6- negative control; 1, 3, 7, & 9- unstained sections on coated slides for immunostaining (but may be used for any stain). Number of slices of SLN shown (a,b,c) is for illustration only and would vary according to the size of the lymph node. (FPTS, formalin-fixed paraffin-embedded tissue sections; H&E, hematoxylin and eosin stain; immuno, immunohistochemistry)

The excised lymph nodes were transected perpendicular to the long axis and processed to make imprint smears as previously reported [35]. The imprint smears were immunostained with the 'MCW melanoma cocktail' using either a manual method or the Dako^® ^Autostainer with a rapid 17-minute immunostaining protocol utilizing the Dako EnvisionTM+ System which does not contain avidin-biotin [35]. One smear of a previously prepared positive control from a known melanoma tumor and processed similar to test smears [35] was also immunostained with each staining batch.

The immunostained imprint smears were examined and interpreted as positive, indeterminate, or negative for melanoma metastases. The time required to go from preparation of imprint smears to evaluation of immunostained smears was also tracked. Based on the algorithm for intraoperative decision, indeterminate interpretations with immunostained imprint smears were considered as negative for statistical analysis.

After imprint smear preparation, the slices of SLNs were fixed in 10% formalin and processed in the usual manner for paraffin embedding. Three FPTS levels, each cut at 200-μm intervals were stained with hematoxylin and eosin (H&E). The levels adjacent to the middle H&E section were immunostained with 'MCW melanoma cocktail' using the avidin-biotin-peroxidase complex (ABC) method described previously [33].

The results were considered positive for melanoma metastases if cytoplasmic immunoreactivity was detected in cell clusters or individual cells that demonstrated morphologic features of melanoma tumor cells. When discrepancies existed between immunostained imprint smears and the immunostained FPTS, the slides were reviewed by another pathologist in an attempt to determine the cause of the discrepancy and to arrive at a conclusion (Table 2).

**Table 2:** Results with rapid intraoperative immunocytochemical evaluation of imprint smears.

Serial No.	No. of SLN per case	SLN		
		
		IC	SP	**FR**^4^
1	2	B-,C-	B-**cn**,C-	N
2	1	A-	A-**cn**	N
3^¶^	2	A1+,A2+,B-	A1+,A2-,B-	**P**
4	1	A-	A-**cn**	N
5	3	A-,B-,C-	A-,B-**cn**,C-	N
6	1	A-	A-	N
7	2	B-,C-	B-,C-	N
8	1	A-	A-	N
9	2	A-, C-	A-, C-	N
10	2	A-,C-	A-**cn**,C-	N
11*	4	**A-**,B-,C-,D-	A+,B-,C-,D-	**P**
12^¶^	4	A-,B+,C+,**D+**	A-,B+,C+,D-	**P**
13^†^	1	**A(Intm)**	A-	N
14	1	B+	B+	**P**
15	1	A-	A-	N
16	3	A+,B-,C-	A+,B-,C-	**P**
17^†^	2	A**(Intm)**,B-	A-,B-	N
18	1	A-	A-	N
19	2	A-,B-	A-,B-	N
20	2	A1-, C+	A1-, C+	**P**
21	1	A-	A-	N
22	2	A-,B-	A-,B-	N
23^1^	^1^2	^1^A1-,^1^B+,^1^C-,D-,E-	^1^A1-,^1^B+,^1^C-,D-,E-	**P**
24	1	A-	A-	N
25	2	A-,B-	A-,B-	N
26	2	A-,C-	A-,C-	N
27	1	B-	B-**cn**	N
28	1	A-	A-	N
29	1	A-	A-**cn**	N
30	1	A-	A-	N
31^2^	4	A-,B-,C-,D-	A-,B-,C-,D-	N
32	2	A-,B-	A-,B-	N
33	1	A-	A-	N
34	4	A-,B-,C-,D-	A-,B-,C-,D-	N
35	3	A-,B-,C-	A-,B-,C-	N
36^3^	1	A-	A-	N
37	2	A-	A-	N
38	2	B-,C-	B-,C-	N
39	2	A-,B-	A-,B-	N
40	3	A-,B-,D-	A-,B-,D-	N
41	2	A-,B+	A-,B+	**P**

*One false negative

^†^Two negative cases showed indeterminate results with IC

^¶^Two SLN were positive unequivocally with IC but negative by SP

^1^Only D&E were submitted as sentinel LN (A,B,C were submitted as non-SLN);

^2^Atypical Spitzoid nevus, recommended SLN biopsy;

^3^Dysplastic nevus, recommended SLN biopsy;

^4^Final results summarizing findings in all SLN in a particular case after immunostaining of permanent sections: total number of positive cases- 8 and negative cases- 33.

*Abbreviations in alphabetical order*: +, positive for melanoma metastases; -, negative for melanoma metastases; **cn**, capsular nevus; FR, final result; IC, rapid intraoperative immunocytochemistry; **Intm**, indeterminate for melanoma metastases; LN, sentinel and non-sentinel lymph nodes; N, negative; **P**, positive; SP, final result on permanent sections.

## Results

The time required for completing the entire procedure from preparation of imprint smears to evaluation of immunostained smears ranged from 24 to 45 (mean, 35) minutes and depended on the size-number of SLNs.

At the case level, melanoma metastases were identified in lymph nodes of 19.5% (8 out of 41) of patients by the immunohistochemical evaluation of FPTS. 87.5% (7 out of 8) of these cases showed metastases in imprint smears immunostained rapidly with the 'MCW melanoma cocktail', potentially saving a second surgery in all these cases. The test demonstrated a sensitivity of 89%, a specificity of 100%, the negative predictive value of 97%, and the positive predictive value of 100%.

At the lymph node level, 12% (9/73) nodes were positive for melanoma metastases with immunostained FPTS. Of these, 89% (8/9) showed melanoma metastases in intraoperatively immunostained imprint smears utilizing the cocktail demonstrating a sensitivity of 90%, a specificity of 100%, the negative predictive value of 99%, and the positive predictive value of 100%).

**Figure 2:**
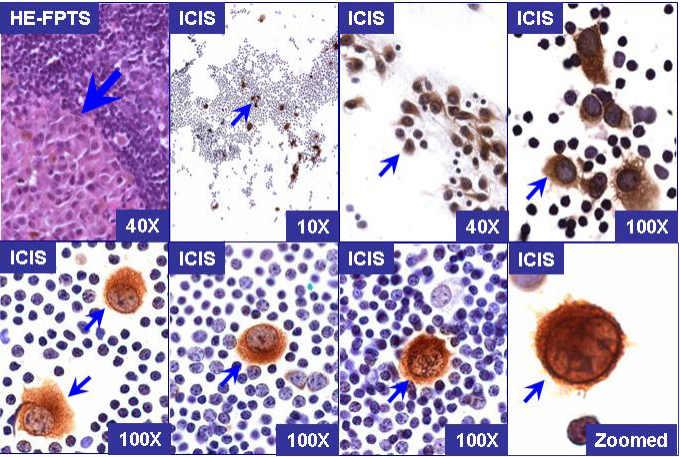
Cytomorphological spectrum of tumor cells (arrows) of melanoma metastases immunostained with 'MCW melanoma cocktail'. The tumor cells were larger and showed high N/C ratio with non-granular cytoplasmic staining around the nuclei with usually clear nuclear details. The nuclear chromatin did not simulate the chromatin of lymphocytes in the background. The nucleoli may be prominent. The cell margins were well defined. (HE-FPTS, Hematoxylin-eosin stained formalin-fixed paraffin-embedded tissue sections; ICIS, immunocytochemical evaluation of imprint smears).

The immunostained tumor cells of melanoma metastases (Figure 2) showed a high nuclear to cytoplasmic ratio with non-granular cytoplasmic staining around the nuclei facilitating easy evaluation of nuclear details [35]. All 7 SLNs from 7 cases with benign capsular melanocytic nevi showed negative results with the intraoperative evaluation of imprint smears.

An immunostained imprint smear was negative in one case with a small focus of melanoma metastases (4 small groups of 4–12 cells each in one small area) in one slice of a single SLN. 2 SLNs showed unequivocal melanoma metastases in rapid immunostained imprint smear (Table 2, case 3 & 12). In these 2 SLNs, metastases were not detected by immunostained FPTS.

Two SLNs from 2 patients (Table 2, case 13 & 17) were interpreted as indeterminate. Both of these SLNs were negative for melanoma metastases by immunohistochemical evaluation of FPTS. In 2 other cases, rare, singly scattered cells with benign morphology but with cytoplasmic immunostaining with the cocktail were observed in both immunostained imprint smears and permanent sections.

## Discussion

In the current study, 8 out of 41 patients showed melanoma metastases in lymph nodes employing immunohistochemical evaluation of FPTS. 7 out of these 8 cases showed metastases on imprint smears immunostained with the 'MCW melanoma cocktail', potentially avoiding a second surgery at a later date in 87.5% of these patients.

On a case basis, rapid intraoperative evaluation of imprint smears with the 'MCW melanoma cocktail' yielded a sensitivity of 89% and a specificity of 100%. On a lymph node basis, sensitivity was 90% and specificity was 100% (Table 2). These encouraging results are more promising than alternative intraoperative approaches such as the frozen-section alone [27], immunostaining frozen sections with a cocktail of Melan- A, HMB-45, & tyrosinase [32], or a morphological evaluation of imprint smears alone [30,38] (Table 3).

**Table 3:** Comparison of studies evaluating melanoma SLNs intraoperatively

Authors	Intraoperative evaluation	Sensitivity (%)	Specificity (%)
Gibbs et al [20A]	FS	43	100
Clary et al [20B]	FS	56	100
Tanis et al [20C]	FS	47	100
Stojadinovic [20]	FS	59	100
Creager et al [21]	IS	38	100
Eudy et al [23]	FS with IHC	86	97
Current study	ICIS	90	100

FPTS, formalin-fixed paraffin-embedded tissue section; FS, frozen section, ICIS, immunocytochemical evaluation of imprint smears with 'MCW melanoma cocktail' (mixture of Melan- A, MART- 1, & tyrosinase); , IHC, immunohistochemistry with cocktail of Melan- A, HMB-45, & tyrosinase); IS, imprint smear; SLNs, sentinel lymph nodes; MM, melanoma metastases.

Finding a positive SLN and completing the regional lymphadenectomy under the same anesthesia offers many advantages. The extra cost for the second procedure was predominantly the hospital cost. In this series with 41 patients, the extra cost (after deducting the cost for the regional lymph node dissection procedure and adding the cost for extended anesthesia) for 8 regional lymph node dissections that needed to be performed at a later date was $ 60,000 (average $ 7,500 per case). In comparison, the cost of the intraoperative immunocytochemical evaluation for 70 SLNs in all 41 cases ([CPT codes 88329 (for intraoperative consult) + 88161 (for preparation and processing of imprint smears for immunostaining) + 88342 (immunostaining and interpretation of immunostained smears)] X 70) was only $33,233, resulting in nearly 50% cost saving.

The most important benefit that cannot be quantified easily is the total avoidance of potential morbidity and mortality associated with an additional surgery at a later date, improving patient care. Additionally, saving the patient from a second visit to the surgical suite and its substantial attendant costs along with a decreased number of office visits (to reschedule the regional lymphadenectomy), lessens the inconvenience for the patient.

In cases with positive intraoperative SLN results, the duration of the surgical procedure would be lengthened for the completion of the regional lymphadenectomy. Though it demands flexibility in surgical scheduling, it may be worth at most institutions.

As observed in one case, immunostained imprint smears of SLN that turn out negative may be followed by positive results after immunostaining the FPTS. This may require the patient to return to the surgical suite for a lymphadenectomy at a later date. The patients should be made aware of this possibility during preoperative consultations.

Due to sampling benefits associated with imprint smears [35], the immunostaining of imprint smears identified metastases that may have been missed by studying immunostained FPTS alone. This was observed in 2 cases in which immunostained imprint smears of 2 SLNs were unequivocally positive for melanoma metastases, but immunostained FPTS were negative (Table 2, case 3 & 12). Additional SLNs submitted in these 2 patients were positive with both immunostained imprint smears and FPTS, further endorsing the true positive nature of finding.

One SLN from another case showed a micro-focus of melanoma metastases in a single slice in immunostained FPTS. However, the cells from this micro-focus with very few tumor cells were not sampled by the imprint smears leading to a false negative result. This underscores the significance of chance factor associated with sampling in general during the evaluation of SLN by any method. Unequivocal positive result, obtained with either immunostained imprint smears or with immunostained FPTS, are significant. In cases where the intraoperative results are positive, SLNs need not be evaluated further by immunohistochemistry on FPTS. However, if the intraoperative result is negative, immunohistochemical evaluation of SLNs should go forward on FPTS. This would enhance the sampling and increase the chances of detecting additional melanoma metastases [35].

As a significant advantage, the cells of capsular nevi in 7 SLNs from 7 cases located in lymph node capsule and/or septa as collections of spindle cells, did not exfoliate and adhere to slides during the preparation of imprint smears. As discussed previously, this is related to the cohesive nature of benign nevus cells in capsular melanocytic nevi, which would not exfoliate cells to the glass slide in contrast to poorly cohesive melanoma cells [33,35]. This result in a distinct advantage for the immunocytochemical evaluation of imprint smears over other methods such as RT-PCR, which may not have the benefit of a morphological correlation to avoid false positive results with benign melanocytic capsular nevi (24).

Two SLNs from 2 patients, negative for melanoma metastases by immunohistochemical evaluation of FPTS, were interpreted as indeterminate in immunostained imprint smears (Table 2 case 13 & 17). Retrospectively, the rare doubtful cells observed in immunostained imprint smears were consistent with mast cells which demonstrated brown staining (even in respective negative controls) due to endogenous peroxidase activity which could not be blocked during the brief endogenous peroxidase blocking step required by the rapid protocol [35]. Familiarity with morphological spectrum of immunostained tumor cells (Figure 1) and other non-specifically stained structures including mast cells in imprint smears prevents indeterminate interpretation [35]. In two other cases, singly scattered cells showing cytoplasmic immunostaining accompanied by small and inconspicuous nuclei were observed. These rare single cells were also present in immunostained permanent sections. The cytomorphology was consistent with a benign interpretation in both immunostained imprint smears and the permanent sections. Similar rare cells with benign morphology have been reported in SLNs evaluated with MART-1 and Melan-A [39].

For various reasons every laboratory may not be comfortable using cytological methods. However, objective nature of interpreting immunostained tumor cells in imprint smears would allow interpretation by most of the pathology laboratories after a minor learning curve [35].

Imprint smears are less expensive and faster than frozen sectioning without cryostat related tissue loss. They avoid the frequently observed problems associated with the frozen sectioning of fatty lymph nodes. If imprint smears are made and the tissue is not frozen, it would prevent the introduction of freezing artifacts and the associated interpretation challenges on the permanent sections of such frozen tissue.

Other approaches for the intraoperative evaluation of SLN have demonstrated lower sensitivities (Table 3) but had 100% specificity, which is extremely important in order to avoid un-indicated regional lymph-adenectomies [26-32]. One study evaluating immunostaining of frozen sections demonstrated a slightly increased sensitivity than when imprint smears or frozen sections alone were used without immunostaining. However, the specificity was less than 100% (Table 3). The immunostained frozen sections may show significant artifacts along with folds, background staining, missing areas, and poor morphology. As HMB45 is known to stain non-melanoma cells in SLN [33,40-42], incorporation of HMB45 in the cocktail used in this study may have contributed further to a reduction in the specificity [32,41,42]. As stated by the reporting authors of the study, a recent consensus group has discouraged frozen-section examination of SLN [32].

In summary, imprint smears, rapidly immunostained with the 'MCW melanoma cocktail' is a sensitive and specific method for the rapid intraoperative evaluation of SLNs. If a SLN is found to be positive for melanoma metastases in this way, the completion of a regional lymphadenectomy during the same surgical procedure for SLN biopsy is feasible. Both negative and indeterminate results should be regarded as negative when considering intraoperative decision making for a regional lymph node dissection during a given surgical procedure. In such cases, the final decision for a regional lymph node dissection should be deferred until the final results with FPTS are available.

## Abbreviations

FPTS, formalin-fixed paraffin-embedded tissue section; H&E, hematoxylin and eosin; SLN, sentinel lymph node;
